# Signaling Pathways Involved in Nutrient Sensing Control in Cancer Stem Cells: An Overview

**DOI:** 10.3389/fendo.2021.627745

**Published:** 2021-03-22

**Authors:** Martha Robles-Flores, Angela P. Moreno-Londoño, M. Cristina Castañeda-Patlán

**Affiliations:** Departamento de Bioquímica, Facultad de Medicina, Universidad Nacional Autónoma de México (UNAM), Mexico City, Mexico

**Keywords:** nutrient sensing, cancer stem cells, mammalian target of rapamycin (mTOR) signaling, adenosine monophosphate-activated protein kinase (AMPK) signaling, hexosamine biosynthesis pathway (HBP) pathway

## Abstract

Cancer cells characteristically have a high proliferation rate. Because tumor growth depends on energy-consuming anabolic processes, including biosynthesis of protein, lipid, and nucleotides, many tumor-associated conditions, including intermittent oxygen deficiency due to insufficient vascularization, oxidative stress, and nutrient deprivation, results from fast growth. To cope with these environmental stressors, cancer cells, including cancer stem cells, must adapt their metabolism to maintain cellular homeostasis. It is well- known that cancer stem cells (CSC) reprogram their metabolism to adapt to live in hypoxic niches. They usually change from oxidative phosphorylation to increased aerobic glycolysis even in the presence of oxygen. However, as opposed to most differentiated cancer cells relying on glycolysis, CSCs can be highly glycolytic or oxidative phosphorylation-dependent, displaying high metabolic plasticity. Although the influence of the metabolic and nutrient-sensing pathways on the maintenance of stemness has been recognized, the molecular mechanisms that link these pathways to stemness are not well known. Here in this review, we describe the most relevant signaling pathways involved in nutrient sensing and cancer cell survival. Among them, Adenosine monophosphate (AMP)-activated protein kinase (AMPK) pathway, mTOR pathway, and Hexosamine Biosynthetic Pathway (HBP) are critical sensors of cellular energy and nutrient status in cancer cells and interact in complex and dynamic ways.

## Introduction

Tumors are not uniform but rather heterogeneous in function. The involvement of stem cell cancer (CSC) subpopulations has been demonstrated in almost all human cancers. These cells have the capacity to replicate the entire tumor and are often denoted as tumor-initiating cells (TICs). They also drive tumor formation, metastatic spread, and relapse, making them a daunting yet promising goal to eliminate cancer (1).

Cancer cells reprogram their metabolic procedures to meet their needs, such as their high proliferation rate: they induce rapid ATP generation to maintain energy status, increase the biosynthesis of macromolecules and induce strict regulation of the cellular redox status. Non-malignant cells obtain ATP, an energy source necessary for survival, from both glycolysis and mitochondrial oxidative phosphorylation (OXPHOS). In contrast, cancer cells mainly get ATP from glycolysis rather than OXPHOS, even in the presence of adequate oxygen concentration (Warburg effect) (2). In a nutshell, most cancer cells depend on glycolysis to generate ATP, even when oxygen is available.

In comparison to glycolysis-based differentiated bulk tumor cells, CSCs exhibit high plasticity showing a distinct metabolic phenotype that can adjust their metabolism to micro-environmental changes depending on the type of cancer by conveniently transferring energy output from one pathway to another or obtaining intermediate metabolic phenotypes (2–4). In either case, the mitochondria’s function is important and focuses on CSC functionality (2). In addition to being a significant source of ATP for cells, mitochondria are involved in the regulation of many signaling pathways in CSCs, such as mitochondrial fatty acid oxidation (FAO) for the generation of ATP and NADPH (2).

Regardless of the primary metabolic phenotype in individual cells, mitochondria often tend to control stemness properties (5–7). Increased mitochondrial biogenesis and mass recognize cells with enhanced self-renewal ability and chemoresistance (7–9), irrespective of the type of cancer. Stem cell mitochondria are smaller in number and display reduced activity relative to differentiated cells (10, 11). All these characteristics result in decreased ROS levels in stem cells (3). The apparent dependency of CSCs, irrespective of their primary metabolic phenotype on mitochondrial function, is a previously unrecognized Achilles’ heel modifiable for therapeutic purposes (2).

The proliferation of cancer cells largely depends on their nutritional surroundings, especially the availability of glucose. It is well-known that CSCs obtain a substantial amount of their energy *via* aerobic glycolysis, which is faster than OXPHOS and far less efficient to generate ATP per unit of glucose consumed, provoking an abnormally high rate of glucose uptake (2). In CSCs, glutamine is also actively absorbed (12). Although the contribution of the metabolic and nutrient-sensing pathways to stemness preservation has been demonstrated, the molecular mechanisms connecting stemness with the nutrient-sensing routes are not well understood (13). However, among these pathways, mTOR and AMPK pathways’ contribution, together with the hexosamine biosynthesis pathway (HBP), is maybe the most significant.

The metabolic phenotype of CSCs has been the focus of extensive study in recent years (2). It is important to emphasize that tumors show cellular heterogeneity. While CSCs prefer glycolysis and have fewer mitochondria, they have high metabolic adaptability that allows them to thrive in conditions of nutrient and stress micro-environmental fluctuations. Using nutrient-sensing pathways such as HBP and those regulated by mTOR and AMPK, stem cells sustain energy output by inhibiting essential processes such as OXPHOS and enhancing others such as glycolysis. In subsequent sections, we will explain how HBP is regulated by the intake of nutrients such as fats, amino acids, and nucleic acids, converting it into a crucial nutrient-sensor for these molecules’ variations. More so, we will describe how the mammalian target of rapamycin (mTOR) and AMP-activated protein kinase (AMPK) pathways participate in nutrient-sensing as a way of regulating cell activity. We will also describe how the interaction among these pathways adjusts the cellular response to nutrients and is essential to stemness maintenance (3).

## Hexosamine Biosynthetic Pathway

**Figure 1:**
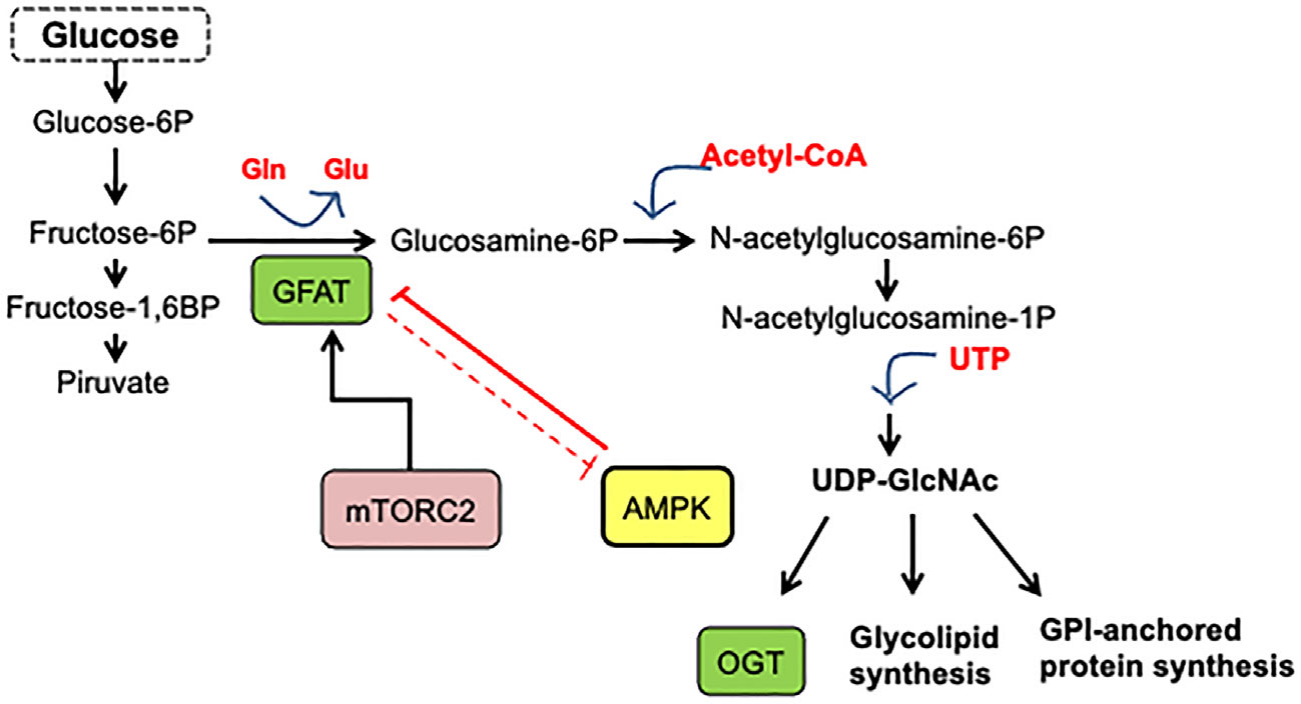
Hexosamine biosynthetic pathway. A fraction (3–5%) of glucose incoming the cell is shunted through the hexosamine biosynthesis pathway. In this pathway, fructose-6-phosphate is converted to glucosamine-6-phosphate by the glutamine/fructose-6-phosphate amidotransferase (GFAT), the gate-keeper enzyme of the route. The main product of the pathway is UDP-GlcNAc, which is the substrate for O-GlcNAc transferase.

Cancer cells obtain a significant amount of energy from aerobic glycolysis, which is faster than OXPHOS but less effective in terms of ATP produced per unit of glucose consumed, resulting in an abnormally high glucose rate uptake. In these conditions, once glucose reaches the cell and is phosphorylated by hexokinase, it can be redirected from the primary glycolytic pathway to secondary pathways. **Figure 1** shows that the flux through the hexosamine biosynthetic pathway (HBP) then increases, resulting in a cellular addiction to glutamine, and glucose is also metabolized through other alternative pathways such as the pentose phosphate pathway (PPP) (2).

Experimental evidence has shown the essential role of HBP in cancer metabolic reprogram and the strong association between cancer progression and enhanced HBP flux (14). Elevated HBP enzyme expression has been reported in many human cancers. HBP is a nutrient-responsive metabolic pathway because by incorporating intracellular glucose, glutamine, acetyl-CoA, and UTP into the synthesis of UDP-GlcNAc, this pathway allows information on the availability of nutrients (13–15). Remarkably, HBP produces the high-energy-donor UDP-GlcNAc, which is the sugar donor utilized for macromolecule glycosylation and the synthesis of other nucleotide sugars. It is also used by O-GlcNAc transferase (OGT) to modify target proteins (13, 14). On the other hand, OGT is also regulated by the input of amino acids, fats, and nucleic acids, making O-GlcNAc a key nutrient-sensor for variations in these macromolecules. **Figure 1** presents the synthesis of UDP-GlcNAc from glucose through HBP. Glutamine/fructose aminotransferase (GFAT) commits glucose to this pathway and represents the gate to HBP. As a result of OGT activity, post-translationally protein modification by O-GlcNAc occurs in the serine or the threonine residues of the target protein.

Accumulating experimental evidence has shown that micro-environmental stress signals in tumors induce phenotypic plasticity and invasion and decide the therapeutic outcome. Since stem cells play a crucial role in the integration of those signals with their self-renewal and maintenance, and with the tissue homeostasis, stem cell behavior is likely regulated directly or indirectly by stress signals to coordinate metabolic stress with an appropriate, tissue-specific response.

The fundamental function of O-GlcNAcylation seems to be the modulation of cellular processes in response to nutrients and cellular stress (16–20). Consistent with this, it has been documented that the inhibition of OGT in colon cancer cells or the incubation of cells under acute nutritional stress that mimics the lack of OGT induces the emergence of an aggressive CD133/CD44 double-positive CSC subpopulation (17).

Although metabolic reprogramming is a characteristic of self-renewing cancer stem cells, very little is known about how their metabolism is regulated to control CSC phenotype. In this regard, it has been demonstrated that the inhibition of the HBP-Hypoxia inducible factor-1 (HIF-1α) axis abrogates glycolysis enhancement and reduces the CSC-like subpopulation (14). Hypoxia-inducible factors (HIFs) are master transcription factors controlling the adaptation of cancer cells to hypoxic conditions often generated in tumors as a consequence of rapid growth. Importantly, it has been demonstrated that HIFs regulate multiple phases of tumorigenesis and are commonly associated in cancer cells with changes in metabolic reprogramming (21). Remarkably, it has been shown that O-GlcNAcylation regulates cancer metabolism and survival stress signaling by regulating HIF-1α signaling pathway (22). In this regard, we have established that *O*-GlcNAc and the activity of OGT are intimately linked with the cell’s nutritional status, as previously reported in several cell systems. Notably, we also found that increased *O*-GlcNAc levels seem to be part of an endogenous stress response associated with cancer cell survival (17). In line with this, our findings have verified that starvation enhances the expression of stem cell markers. Still, importantly, it validates the perception that the OGT activity and HBP pathway are closely integrated with the nutritional status of the cells and contributes to the regulation of stemness maintenance (17).

## The Mammalian Target of Rapamycin Pathway

**Figure 2:**
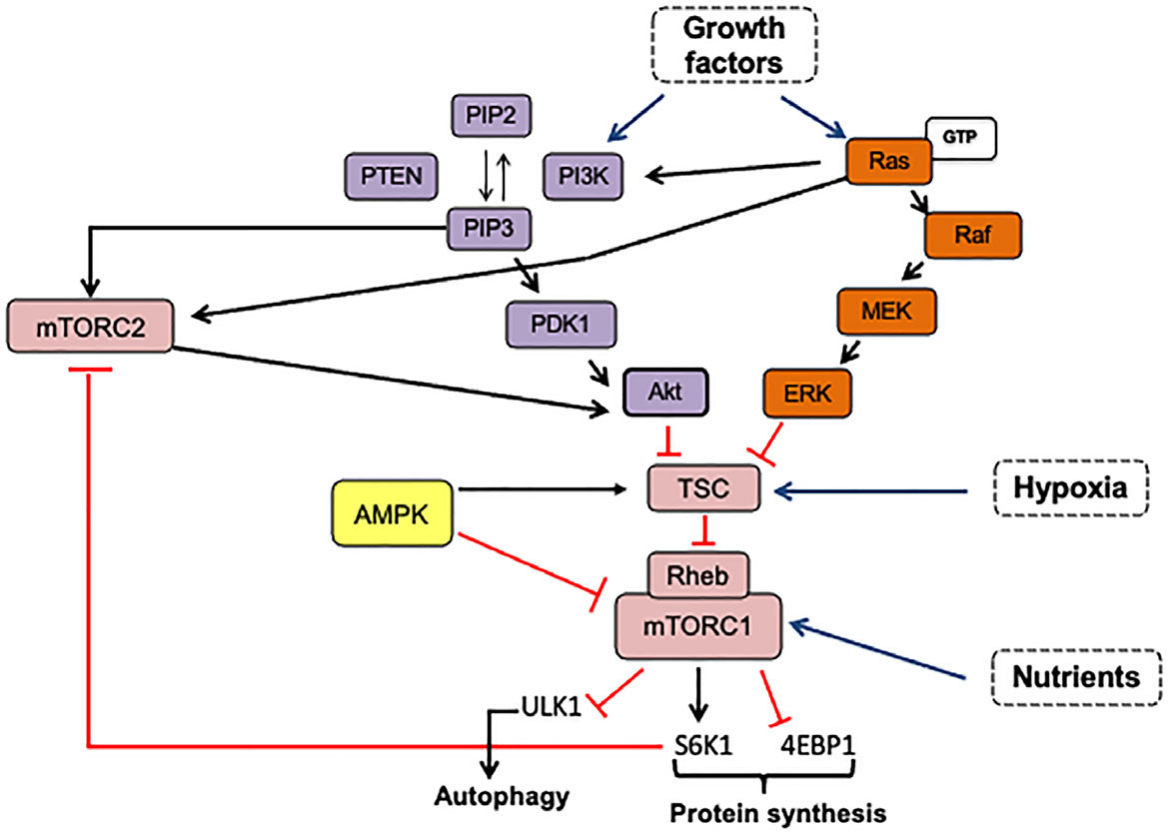
mTOR is activated by growth factors *via* PI3K-Akt and RAS/MAPK pathways. The phosphorylation of phosphatidylinositol 4,5 biphosphate (PIP2) is catalyzed by PI3K producing PIP3. Once PIP3 is formed, it induces the recruitment of proteins with PH domain such as PDK1, Akt, and mTORC2 complex, facilitating Akt-Thr308 and Akt-Ser473 phosphorylation by PDK1 and mTORC2, respectively. Activated Akt inhibits the TSC complex and promotes mTORC1 activation by Rheb-binding. The Ras/AMPK pathway can regulate both mTOR complexes *via* the Ras-Raf-MEK-ERK signaling cascade. Activated ERK inhibits the TSC complex by direct phosphorylation, and Ras-GTP can bind to mTORC2, increasing its kinase activity. mTORC1 activation in response to amino acids can be dependent or independent on Rag GTPases. Also, mTORC1 can control its own activation and mTORC2 activity. mTORC2 is negatively regulated by mTORC1-S6K1 that phosphorylates mSN1 and Rictor. The arrows indicate: →, activation signals; ┴, inhibition signals; → pathway activators.

**Figure 3:**
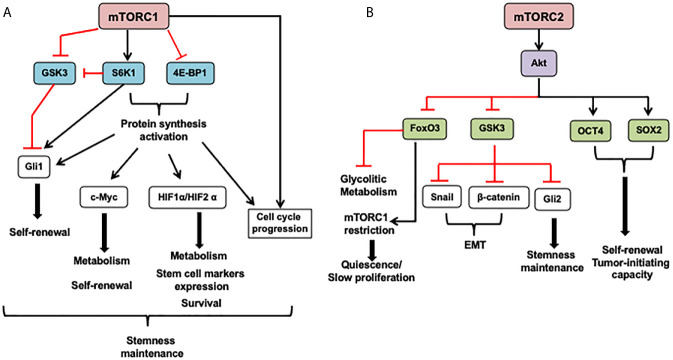
mTOR in cancer stem cells (CSCs). **(A)** The mTORC1 can modulate cell metabolism, cell survival, proliferation, and stem cell maintenance mostly through protein synthesis activation of transcription factors that induce the expression of genes coding proteins involved in these functions. Also, mTORC1 can promote Gli1 (downstream effector of the Hedgehog pathway), nuclear localization through its effector S6K1 that induces Gli1 releasing from its endogenous inhibitor, SuFu, and inhibits GSK3-mediated its degradation. Besides, mTORC1 under mitogenic signals and amino acid availability controls GSK3 nuclear import and, in turn, its nuclear functions as mediating c-Myc degradation. **(B)** The role of the mTORC2 complex in CSCs is mainly mediated by its effector Akt. This protein can phosphorylate many substrates, including OCT4 and SOX2, transcription factors that regulate stem cell self-renewal and promote pluripotency. Akt directly phosphorylates these transcription factors increasing its stability and triggering its nuclear import. Also, Akt inhibits GSK3, leading to GSK3 substrates stabilization such as β-catenin and Snail implicated in epithelial-mesenchymal transition (EMT) and Gli2 that increases SOX2, OCT4, and Nanog expression in CSCs. FoxO3 inhibition by mTORC2 signaling through Akt activation and HDACs inhibition avoid FoxO3 nuclear localization and FoxO3 deacetylation, respectively. It might release FoxO3-induced c-Myc repression, promoting upregulation of glycolytic metabolism. 4E-BP1, eukaryotic translation initiation factor 4E binding protein 1; FoxO3, forkhead box O3; GSK3, Glycogen synthase kinase; Gli1/2, Glioma-associated oncogene; HIF1 α, hypoxia-inducible factor 1 subunit alpha; S6K1, ribosomal protein S6 kinase; OCT4, octamer-binding transcription factor 4; SOX2, RY-box transcription factor 2. SuFu, SUFU negative regulator of hedgehog signaling. The arrows indicate: →, activation signals; ┴, inhibition signals.

The mTOR pathway combines extrinsic and intracellular signals to control cellular processes such as proliferation, cell survival, metabolism, cytoskeleton organization, and autophagy (**Figure 2**). It functions as a nutrient and growth factor sensor as well as a stress sensor in normal and cancer cells. Additionally, this pathway’s fine-tuning regulation contributes to the precise equilibrium between self-renewal and differentiation of stem cells (**Figure 3**) (23, 24).

The activation of the Phosphatidyl-inositol-3 kinase (PI3K)/Akt) or the Ras/ERK signaling pathways by growth factors results in mTOR activation, as shown in **Figure 2**. The dysregulation of these pathways is very common in many cancer types: Receptor Tyrosine Kinases (RTKs) amplification and mutations, PIK3CA or Ras mutations, and loss-of-function mutations in negative regulators such as PTEN, collaborate to constitutively activate either PI3K/Akt or Ras/ERK signaling coupled to mTOR signaling.

### Structure and Regulation of Mammalian Target of Rapamycin

The mTOR protein is a serine/threonine kinase that operates through two distinct multiprotein complexes, mTOR complex 1 (mTORC1) and mTOR complex 2 (mTOR complex 2). These complexes share components such as the positive-regulator mammalian lethal with SEC13 protein 8 (mLST8), the negative-regulator DEP domain-containing protein 6 (DEPTOR), and Tti1/Tel2 complex involved in mTOR complex stability and assembly (25). Other components, such as the regulatory-associated protein of mTOR (Raptor) and negative-regulator proline-rich Akt substrate 40 kDa (PRAS40), are just part of the mTORC1 complex. Raptor recruits mTORC1 substrates, such as ribosomal S6 kinase (S6K) and eukaryotic translation initiation factor 4E-binding protein 1 (4E-BP1) to promote protein translation and general anabolic metabolism (26). The mTORC2 complex includes the unique rapamycin-insensitive mTOR (Rictor) companion proteins, the mammalian stress-activated MAPK interacting protein 1 (mSin1) regulatory subunit, where Rictor and Sin1 recruit independent substrates to mTORC2-activated, including SGK1, Akt, and PKC (27, 28).

MTOR complexes have various roles within cells in response to extra and intracellular signals, such as oxygen levels, energy stress, nutrients, and increased availability of growth factors. In the presence of nutrients and energy, mTORC1 acts as a master regulator of cell growth and metabolism by fostering anabolic processes. mTORC1 translocates to the lysosome membrane in response to a stimulus, where the binding of Rheb activates mTOR (29, 30). The Rheb-GTP induces a conformational change that accelerates its kinase activity. mTORC1 activation is modulated upstream by the tuberous sclerosis complex (TSC1/TSC2), which has GAP activity that accelerates the transition from Rheb-GTP to inactive Rheb-GDP. Regulation of Rheb-mTORC1 binding integrates mTORC1 activation/inhibition to multiple upstream signals (23, 24). For instance, as shown in **Figure 5**, the PI3K-Akt pathway and MAPK/Ras inhibit TSC in response to growth factors and insulin, increasing mTORC1 activity. AMPK can suppress mTORC1 activation through TSC2 phosphorylation increasing its GAP activity or through Raptor phosphorylation and dissociation from mTOR, resulting in the biosynthesis process deactivation in response to energy deficit (31, 32).

Like mTORC1, mTORC2 activation can be through dependent/independent growth factors that could be closely linked to its subcellular localization (33). As it can be seen in **Figure 5**, growth factor-PI3K signaling seems to activate mTORC2 independently on the TSC1/2-Rheb axis. One of the mechanisms suggested is that PI3K-generated PIP3 binds to the PH domain within the mSin1 subunit, consequently varying the mTOR conformation and exposing its catalytic domain. Then, mTORC2-bound to the plasma membrane can phosphorylate Akt to maximally stimulate its catalytic activity (34). Also, partially activated Akt by PKD1 can phosphorylate mSin1 and then enhance mTORC2 activation. Activated-mTORC2 phosphorylates Akt at Ser473 to fully activate it in response to growth factors stimulation (35). mTORC2 can also phosphorylate Akt on Thr450 and PKCα on Thr638, regardless of growth factor stimulation. Although there is a less nutrient-sensing mechanism associated with mTORC2 activation, its crosstalk regulation with mTORC1 may be a way to induce mTORC2 activation under nutrient starvation and energy stress. mTORC1-S6K modulates mTORC2 activation through phosphorylation of mSN1 and Rictor subunits (36, 37). However, Kazyken et al. (38) showed that AMPK directly phosphorylates mTORC2 and increases its catalytic activity independently of mTORC1-mediated negative feedback as a mechanism to enhance cell survival under acute energetic stress conditions (**Figure 5**).

Ras/MAPK signaling pathway positively regulates mTORC1 signaling *via* extracellular signal-regulated kinase (ERK)- and p90 ribosomal S6 kinase (RSK), inactivating by phosphorylation TSC2, or by phosphorylating Raptor (39) (**Figure 5**). Likewise, Raptor can regulate the Ras/MAPK pathway through competitive binding with Ras for SHOC2 leucine-rich repeat scaffold protein (SHOC2), resulting in Ras/MAPK inhibition. But also SHOC2 inhibits Raptor-mTOR interaction, mTORC1 activity, and turning out to trigger autophagy (40). This negative crosstalk allows precise control between proliferation and survival signals. As it can be observed in **Figure 5**, the mTORC2 complex is also susceptible to regulation by oncogenic Ras. In this respect, Kovalsky et al. (41) identified the physical interaction between Ras and mTORC2 complex at the plasma membrane to increase mTOR kinase activity. Thus, disruption of mTORC2-Ras association impairs Ras-driven tumor growth, migration, and invasiveness (41, 42).

Cancer cells are commonly exposed to hypoxia and nutrient depletion. These conditions, along with other stressful conditions such as hyperactive metabolism, mitochondrial dysfunction, and chronic oncogenic mTOR activation, force them to adapt to survive. Besides, they often correlate with other cellular stresses such as endoplasmic reticulum (ER) stress and oxidative stress. Because cancer cell growth is dependent on ATP-demanding anabolic processes, such as lipid, protein, and nucleotide biosynthesis, they are likely to benefit from mTORC1 activation, which promotes building block biosynthesis and thus contributes to abnormal proliferation. It should be considered, however, that mTORC1 inhibits oncogene Akt through negative feedback loops. Thus, persistent mTORC1 activation results in Akt inhibition and thus induces apoptosis. As a result, cancer cells need to balance mTORC1 activity to keep biosynthetic processes and Akt active simultaneously (43). On the other hand, activation of mTORC1 by nutrients and growth factors also leads to autophagy inhibition through the phosphorylation of multiple autophagy-related proteins involved in autophagy initiation and autophagosome nucleation (23, 30). Since mTORC1 is a potent autophagy inhibitor, it seems paradoxical that cancer cells’ survival also requires simultaneous mTORC1 and autophagy activation. This fact again indicates that cancer cells need to maintain a delicate balance between mTORC1 activity and survival mechanisms such as autophagy to benefit from both. Consistent with this, cancer cells have evolved protective tools to avoid the induction of cell death by chronic stresses. Examples of such mechanisms are metabolic reprogramming (the Warburg effect), increased glucose uptake, antioxidant protein synthesis, autophagy and angiogenesis induction, and stress granule formation. There are also activating and inhibiting inputs on the mTORC2 network during different stresses. Examples of them are the activation of mTORC2 by hypoxia (43), and the inhibition of mTORC2 by ER stress (44), and by oxidative stress (45, 46).

### Mammalian Target of Rapamycin Regulation in Cancer Stem Cells

The mTORC1 complex can modulate cell metabolism, cell survival, self-renewal, and stem cell maintenance mostly through protein synthesis activation of transcription factors that induce the expression of genes coding proteins involved in these functions, as it is depicted in **Figure 3**. Under mitogenic signals and amino acid availability, mTORC1 controls GSK3 nuclear import and, in turn, its nuclear functions, such as mediation of c-Myc degradation (47). Besides, mTORC1 can promote Gli1 (downstream effector of the Hedgehog pathway) nuclear localization through its effector S6K1 that induces Gli1 releasing from its endogenous inhibitor, SuFu, and inhibits its GSK3-mediated degradation (47).

The accumulating experimental evidence has shown that cellular metabolism differences between CSCs may depend on the microenvironment and dysregulation of intracellular pathways that control metabolism. The mTOR nutrient-pathway sensor has a critical role in CSCs metabolic plasticity. It must be considered that the impact of mTOR inhibition in stemness can be explained partly by mTOR signaling-mediated regulation function over the pluripotent transcriptional factors, but also by its crosstalk with pathways involved in self-renewal such as Notch, Hedgehog, and Wnt signaling.

The mTORC2 role in CSCs is mainly mediated by its effector Akt (**Figure 3**). This protein can phosphorylate many substrates, including OCT4 and SOX2 transcription factors involved in stem cell self-renewal and multipotency promotion. Akt directly phosphorylates these transcription factors increasing its stability and triggering its nuclear import. Also, Akt inhibits GSK3, leading to GSK3 substrates stabilization such as β-catenin and Snail implicated in epithelial-mesenchymal transition (EMT), and Gli2 that increases SOX2, OCT4, and Nanog expression in CSCs. On the other hand, FoxO3 inhibition by mTORC2 signaling through Akt activation and HDACs inhibition avoid FoxO3 nuclear localization and FoxO3 deacetylation, respectively. It might release FoxO3-induced c-Myc repression, promoting upregulation of glycolytic metabolism (48). However, FoxO3 plays a relevant role in restrain mTORC1 overactivation, enhance survival, and promote stem cell quiescence (49, 50).

As explained before, mTOR hyperactivation is common in tumor bulks. However, this hyperactivation also occurs in the subpopulation of CSCs. PI3K/mTOR activation plays a significant role in maintaining cancer stem-like cells for *in vitro* colony formation ability, spheres formation capacity, and *in vivo* tumorigenicity (51–53). In comparison, mTOR signaling inhibition appears to be necessary to maintain quiescent leukemia stem cells’ reservoir population. At the same time, its activation is needed in the cycling of leukemia progenitors and the stem population’s activation during leukemogenesis (54). Boral et al. (55) identified mTORC2 as crucial signaling in the long-term dormancy maintenance and survival of circulating tumoral cells and bone marrow resident breast cancer cells.

The pluripotency factors SOX2 and OCT4 can modulate PI3K/Akt signaling through transcriptional regulation. While SOX2 supports PI3KCA gene expression, OCT4 represses Akt1 transcription (56, 57). In turn, Akt can phosphorylate both pluripotency transcription factors, increasing their protein stability and subcellular localization (57–59). Akt-mediated OCT4-T235 phosphorylation prevents the repression of Akt1 promoter and favors its transcription, and beyond leads SOX2-OCT4 binding and transcription of stemness genes containing SOX2-OCT4 binding motifs in embryonal carcinoma cells (60). PI3K/Akt/mTOR inhibition reduces SOX2 and OCT4 protein levels and thereby self-renewal and tumor-initiating capacity in some cancers (61–63). Dual PI3K and mTOR inhibitors decrease the sphere-forming ability (64), EMT proteins, and CSC markers expression. They also increase the radiosensitivity in prostate cancer cells (65), and inhibit the self-renewal capacity, tumor growth and promote the differentiation of glioblastoma CSCs (65, 66) and Colorectal CSCs (67). However, both mTOR complexes inhibition, rather than mTORC1 and PI3K inhibition, depletes SOX2 and OCT4 protein levels in glioblastoma cells and suppresses the ability to form tumor-spheres as well (68). Altogether this evidence points out mTORC, particularly mTORC2, with a relevant role in stemness maintenance likely through Akt phosphorylation.

It has been reported that mTOR plays an essential role in radiotherapy and chemotherapy resistance through induction EMT phenotype, an increase of stem cell marker expression, and spheres-formation efficiency. Consistent with this, PI3K/mTOR inhibition reverses this phenotype (64, 69, 70). Besides, it has also been reported that mTOR plays a role in the development of drug resistance through a dependent or independent metabolism adaptation in cancer stem cells in response to chemotherapy or other stressors. Although PI3K/mTOR dual inhibitors have been used to reduce CSCs maintenance and function as mentioned above, cells treated with this class of drugs also have shown resistance in part for induction of compensatory activation of other signaling cascades.

## Adenosine Monophosphate-Activated Protein Kinase Pathway

A vital sensor of cellular energy and nutritional status in eukaryotic cells is adenosine monophosphate (AMP)-activated protein kinase (AMPK). In addition to these canonical roles, to facilitate cell survival, AMPK plays a significant role in controlling mitochondrial respiration, nutrient transport, autophagy, differentiation, longevity, and cell polarity (71).

**Figure 4:**
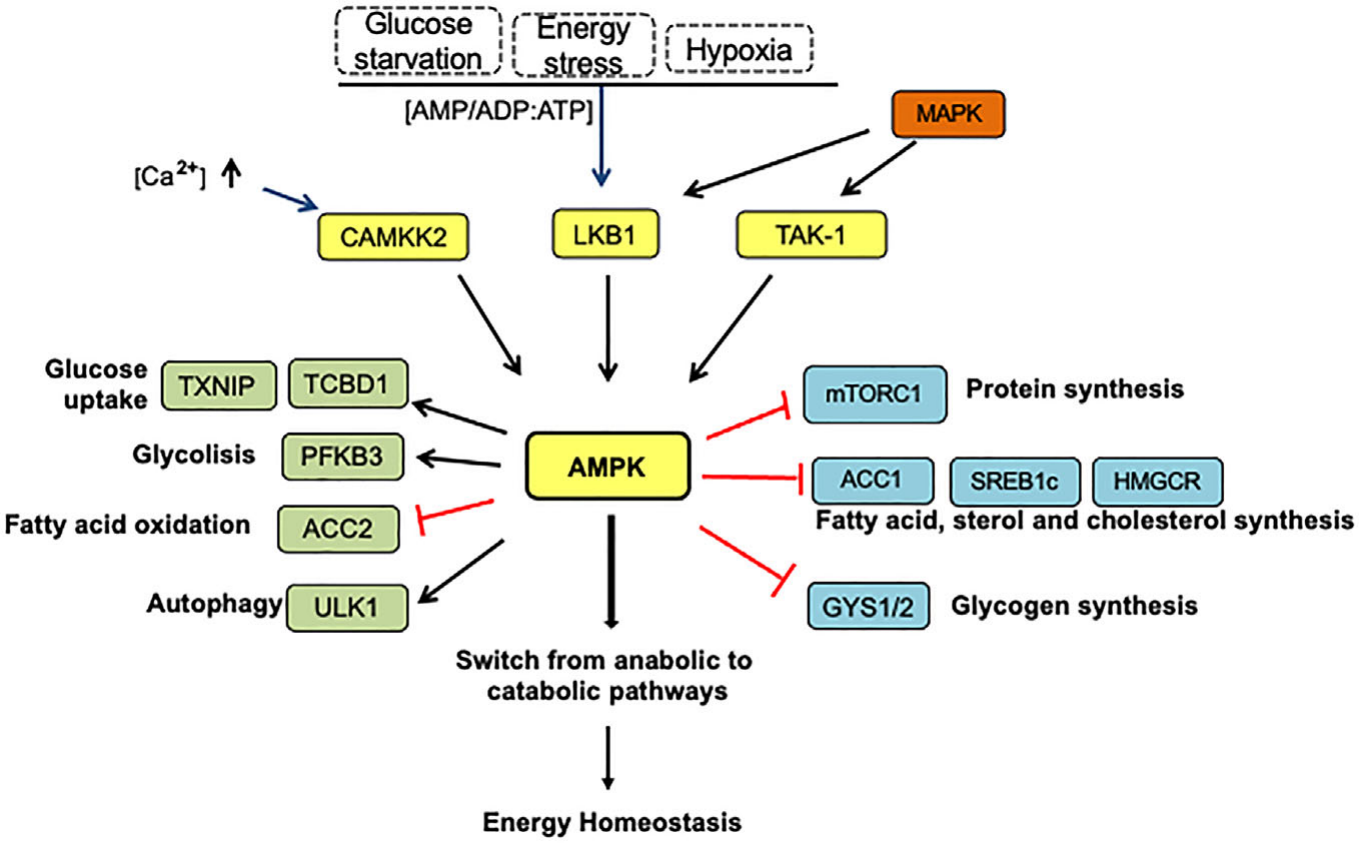
The AMPK pathway activation and energy homeostasis. Under energy stress, AMPK is phosphorylated at Thr 172 by LKB1 in response to variations in AMP: ADP/ATP ratios. Other upstream kinases such as calmodulin-dependent protein kinase kinase 2 (CAMKK2) activated by intracellular calcium and transforming growth factor-β-activated kinase (TAK1) represent alternative AMPK activation forms. In this context, AMPK-activated can repress anabolic processes and increase catabolism to restore energy balance. AMPK suppresses the ATP-consuming anabolic pathways by direct phosphorylation and inhibition of several proteins: mTORC1, acetyl-CoA carboxylase (ACC1), SREBP (sterol response coactivator), HMGCoA reductase (HMGCR), which play critical roles in protein, fatty acid, sterol, and cholesterol synthesis, respectively. AMPK prevents glycogen storage by inhibitory phosphorylation of the glycogen synthases (GYS1 and GYS2). In addition, AMPK also stimulates the catabolic pathways to produce ATP by several mechanisms. First, increasing glucose utilization by phosphorylation and inactivation of domain family member 1 (TBC1D1) and thioredoxin-interacting protein (TXNIP), which control the translocation of glucose transporters GLUT4 and GLUT1 to the plasmatic membrane, respectively. Second, AMPK increases glucose flux along the glycolytic pathway by PFKFB3 (6-phosphofructo-2-kinase/fructose-2,6-biphosphatase 3) phosphorylation, which affects the PFK1 activity, a rate-limiting enzyme in glycolysis. AMPK indirectly stimulates fatty acids transport into the mitochondria by ACC2 inhibition, in turn promoting fatty oxidation. On the other hand, AMPK induces autophagy directly by ULK1 phosphorylation, a kinase essential for autophagy, and indirectly by mTORC1 inactivation. The arrows indicate: →, activation signals; ┴, inhibition signals.

AMPK becomes activated in response to energy stress resulting from reduced production of ATP (e.g., low glucose, starvation, oxidative stress, hypoxia) or excessive intake of ATP (e.g., cell proliferation, muscle contraction, anabolism) (72). By competitively binding both species, AMPK detects changes in the AMP/ATP ratio, resulting in its phosphorylation by upstream kinases and differential regulation of several downstream targets that govern anabolic and catabolic pathways (**Figure 4**) (73). Dysregulation of energy homeostasis is considered a significant driver of changes in many human diseases such as obesity, type 2 diabetes, and cancer (74).

### Adenosine Monophosphate-Activated Protein Kinase Structure

AMPK is a heterotrimeric complex consisting of catalytic α subunits and regulatory β and γ subunits. The genomes of virtually all eukaryotes contain genes that encode at least one of these subunits. Mammalian cells have genes encoding α1/α2 isoforms (PRKAA1/PRKAA2), encoding β1/β2 isoforms (PRKAB1/PRKAB2, and encoding γ1, γ2, γ3 isoforms (PRKAG1/PRKAG2/PRKAG3), which can form 12 distinct heterotrimeric combinations (71). Individual AMPK subunits in humans and mice exhibit significant differences in tissue-specific expression, subcellular localization, and subunit association. While it is understood that isoforms are commonly expressed in most cells, there is a favored isoform combination in given tissue response to a variation in cell physiology (75).

AMPK complex formation, activity, and substrate phosphorylation are affected by cancer (76). The N-terminal α subunit includes a protein kinase domain (KD) linked to a C-terminal, which is required for binding β and γ subunits. The α domain is a typical serine/threonine kinase, which can be phosphorylated by an upstream kinase and enhance its activity more than 100-fold (76). The α-KD is immediately followed by an auto-inhibitory domain (α-AID) that maintains the α-KD in an inactive conformation in the absence of AMP. The α-AID is linked to the globular C-terminal domain (α-CTD) by a flexible regulatory segment (α-linker), which plays a crucial role in the allosteric activation of AMPK by AMP (76).

The β subunit is N-terminally myristoylated at Gly2 (77). A carbohydrate-binding module (CBM), sometimes called the glycogen binding domain (GDB), is located in the central β subunit, which senses the cellular energy in the form of glycogen. The C-terminal region of the β subunit acts as a scaffold to enable the binding of the α and γ subunits (77).

Four cystathionine-β-synthase (CBS 1-4) domains make up the γ subunit involved in the nucleotide-binding (AMP, ADP, or ATP). However, only three sites contribute to nucleotide regulation depending on the cellular energy status (77, 78). Although CBS2 is empty because it is not competent to bind any of the adenine nucleotides, CBS4 retains high-affinity AMP binding and is identified as a “non-exchangeable” site (75).

### Adenosine Monophosphate-Activated Protein Kinase Regulation

AMPK becomes activated in response to energy stress by increasing ADP and AMP cellular levels due to ATP consumption. The enzyme’s allosteric activation is provided by the AMP and other small-molecule activators that bind to the Δ subunit (79, 80). AMPK is also activated by a great variety of natural and synthetic small molecules (75), and these stimuli have different effects on AMPK depending on the isoform combination of the complex (74). The conformational change in the catalytic α subunits of the AMPK enables the phosphorylation of a conserved threonine 172 by upstream kinase Liver Kinase B1 (LKB1) to increase the AMPK activity by >100-fold. As a result, AMP inhibits the dephosphorylation of Thr-172 in AMPK, which switches to catabolic pathways that produce ATP (such as glycolysis and autophagy) and inhibits the use of ATP (anabolic processes such as protein and fatty acid biosynthesis) (81).

Furthermore, as shown in **Figure 4**, AMPK can be activated in mammals by calcium/calmodulin-dependent protein kinase- kinase β (CaMKK) during intracellular Ca^2+^ release when no changes in nucleotides are detected (82). The transforming growth factor-β-activated kinase (TAK-1), usually considered to act upstream in MAPK (mitogen-activated protein kinase) pathways, can also activate AMPK by its phosphorylation at Thr172. The physiological role of this, however, remains uncertain (82).

In addition to Thr172, crucial roles are played by other sites in AMPK activity: α1 at Ser173 and α1Ser485 residues participate in the negative regulation of AMPK by PKA (cAMP-dependent protein kinase). Moreover, phosphorylation of AMPKα at Ser485/491 due to cells’ stimulation with insulin causes decreased AMPK activity (81).

### Adenosine Monophosphate-Activated Protein Kinase and Cancer Stem Cells

Activation of AMPK is a crucial mechanism that supports tumor cell survival because cancer cells are metabolically adapted to survive, particularly under nutrient or energy stress conditions. AMPK activation promotes cell survival and cell growth within tumors that undergo depletion of catabolic substrates by facilitating the transition from anabolic to catabolic metabolism by inhibiting anabolic programs and mTORC1 signaling (83, 84). Consistent with this, many genetic approaches have demonstrated that AMPK promotes cancer cell survival, proliferation, and migration by redox homeostasis in malignant cells cooperating with oncogenes such as c-Myc. This cooperation results in increased cell transformation, metabolic reprogramming, regulation of microtubule dynamics, and provide protection against chemotherapy and radiation (76).

The roles played by AMPK in stem cells, what are the metabolic conditions under it is most important, and which are the substrates that mediate its activity in stem cells remain to be determined to a great extent (85). Cancer stem cells tend to be mainly dependent on glycolysis for ATP production. While AMPK’s role in controlling cell metabolism and energy status is significant, one would expect it to have implications for the maintenance of stem cells. Although the value of AMPK has not yet been extensively studied in stem cells (85), since AMPK is active when the cell energy conditions are low, and given the high-energy demands of cell division, it seems counterintuitive that it promotes stem cell self-renewal. AMPK activity may ensure the completion of mitosis under low energy conditions since cell cycle arrest at this level could have catastrophic implications for a cell’s genomic stability.

It has recently been reported that in response to glucose restriction stress, activated AMPK can translocate into the nucleus with pyruvate kinase M2 (PKM2) isoform through Ran protein. Nuclear PKM2 binding to Oct4 can upregulate cancer stemness-related genes (CD133, CD44, LDHA, NANOG), thus promoting the CSCs population’s enrichment from several human cancer types (86).

In response to energy stress, AMPK inhibits cellular protein biosynthetic pathways and enhances autophagy, a catabolic mechanism that recycles intracellular nutrients to sustain cell survival under nutrient-deprived conditions (87). Under energy stress conditions, AMPK activates autophagy phosphorylating the autophagy regulator ULK1, while inhibits mTORC1 phosphorylating Raptor and the TSC1-TSC2 complex (88). AMPK-mediated autophagy activation allows CSCs to survive in the tumor microenvironment under low levels of both oxygen and nutrient levels. Autophagy also promotes the expression of stem cell markers such as CD44 and spheroid formation (89).

Several pathways that regulate metabolism and autophagy had been used as targets in cancer therapy. Metformin, a drug commonly used to treat type-2 diabetes, is considered a potential anticancer therapeutic agent of CSCs because it inhibits ATP production by inhibiting the mitochondrial electron transport chain. Interestingly, recent data demonstrate that metformin is an AMPK activator and mTOR inhibitor that suppresses CSCs in some cancers (90). Besides, Kim et al. (91) recently reported that glutamine metabolism also plays an essential role in regulating the sensitivity of colorectal CSCs to metformin and that this occurs by an AMPK/mTOR pathway-dependent mechanism.

It has been suggested that AMPK possesses tumor suppressor-like activity because, in response to energy stress, it is activated by the upstream kinase LKB1, a tumor suppressor whose function is often lost in human cancers (92). But unlike LKB1, α, β, and γ subunits of AMPK are rarely mutated in human cancers and are actually amplified (93).

## Interplay Between Hexosamine Biosynthesis Pathway, Mammalian Target of Rapamycin, And Adenosine Monophosphate-Activated Protein Kinase Pathways

The Hexosamine Biosynthetic Pathway is a nutrient-responsive metabolic pathway that generates the OGT substrate, UDP-GlcNAc. Approximately 3-5% of cellular glucose deviates to this pathway as well as glutamine, acetyl-coenzyme A (CoA), and uridine (**Figure 1**) (94). O-linked β-N-acetylglucosaminylation (O-GlcNAcylation) is a very dynamic post-translational protein modification. The modification occurs on serine (Ser) or threonine (Thr) residues of multiple nuclear, cytoplasmic, and mitochondrial proteins. Two enzymes participate in the modification process: while OGT (O-GlcNAc transferase) transfers the N-acetylglucosamine (GlcNAc) residue from UDPGlcNAc to the target proteins, OGA (O-GlcNAcase) removes it from proteins. OGT and OGA-mediated O-GlcNAc cycling disturbances constitute a significant force for aberrant cell signaling in cancer. Indeed, several studies have shown that cancer-related glucose metabolism dysregulation correlates with a rise in OGT expression and global levels of OGlcNAcylation in malignant cells (17).

**Figure 5:**
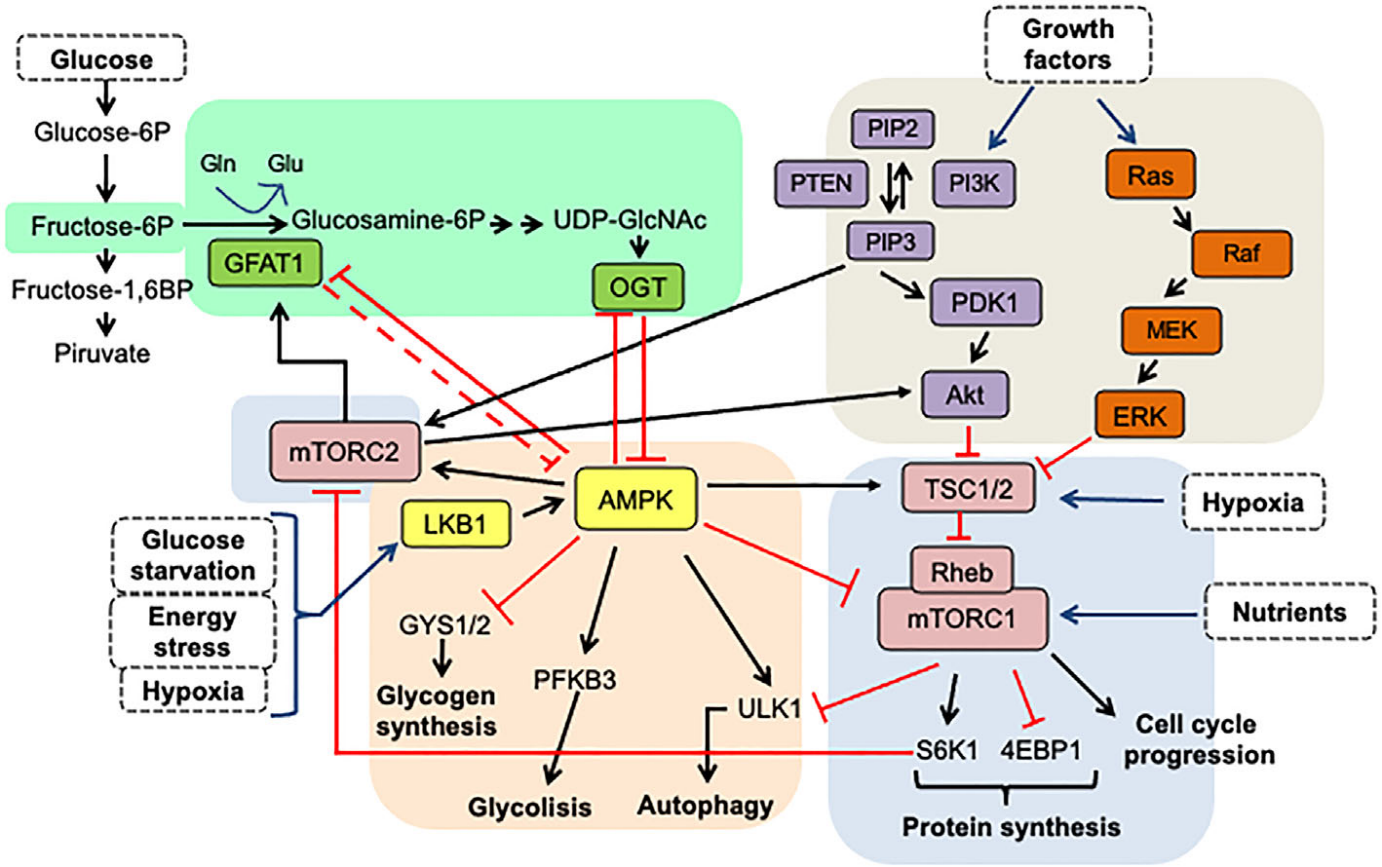
The interplay between HBP, mTOR, and AMPK signaling pathways. The HBP pathway senses glucose, glutamine, and nucleotide levels to produce UDP-GlcNAc, the primary metabolite for protein O-GlcNAcylation, *via* the enzyme OGT. GAFT1 and OGT suppress the AMPK activity, which is a master energy stress sensing enzyme. AMPK activated by phosphorylation favors the processes that produce ATP over the biosynthesis of molecules. Therefore, AMPK functions as a negative regulator of mTORC1, which induces translation and promotes cell growth when there are high levels of nutrients. Furthermore, AMPK activates TSC1/2, which ensures complete suppression of mTORC1 activity. Also, AMPK and mTORC1 pathways feedback take over the ULK1 activation, a protein necessary for the induction of autophagy. On the other hand, growth factors activate the AKT and MAPK kinase pathways, which convergence in the same way in the inactivation of the TSC1/2 complex, the negative regulators of mTORC1. On the other hand, the mTORC2 complex activity is controlled by glucose and acetate levels through acetyl-CoA, an intermediary metabolite in glycolysis, fatty acid catabolism, and the HBP pathways. mTORC2 also converges with the HBP pathway in the stimulation of GFAT1. In consequence, the interplay between these signaling pathways is involved in nutrient sensing as a means of regulating cell activity and growth and, more importantly, in reacting to changes in the microenvironment of the tumor. The arrows indicate: →, activation signals; ┴, inhibition signals.

**Figure 6:**
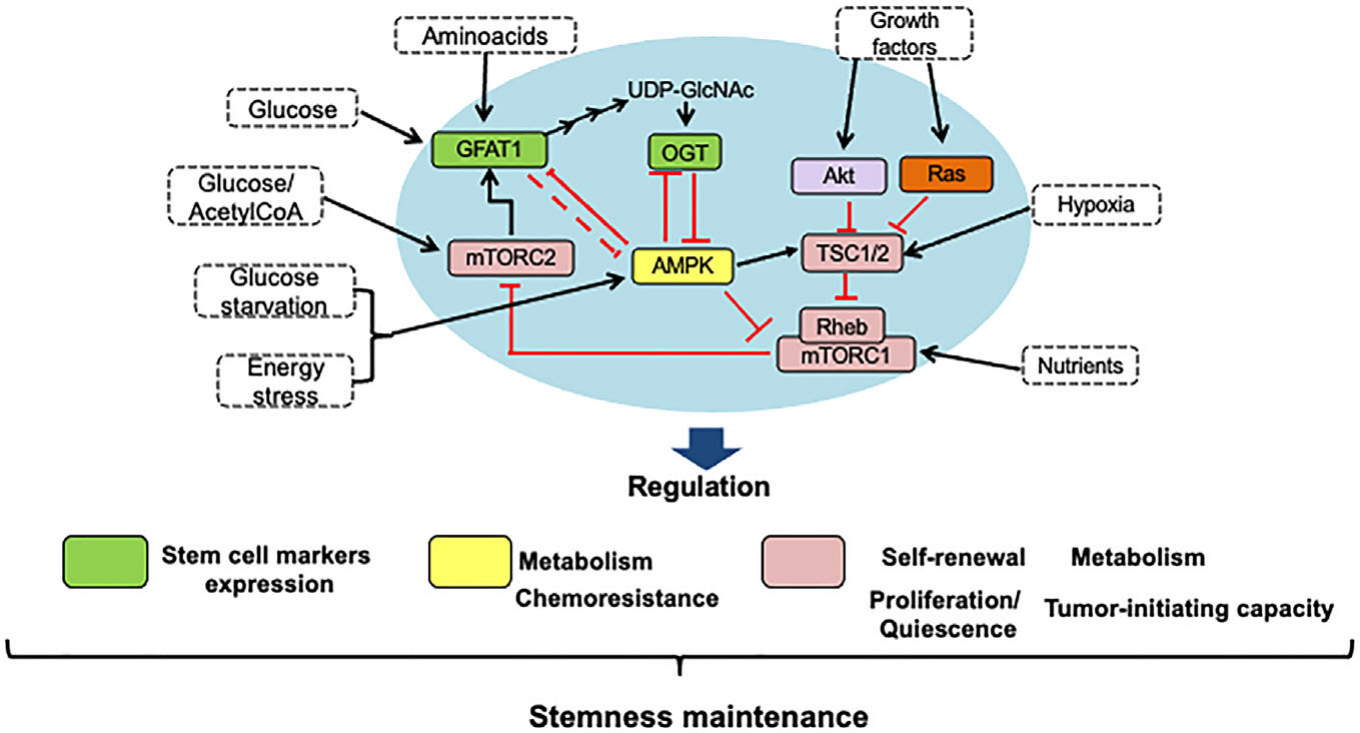
HBP, mTOR, and AMPK signaling pathways interaction is crucial to stemness maintenance. Using nutrient-sensing pathways such as HBP, mTOR, and AMPK, stem cells respond to nutritional cues, and the crosstalk between them is key to maintaining stemness. Thus, to regulate the maintenance of stem cells in the tumor microenvironment, conditions such as the availability of nutrients, growth factors, and oxygen can modulate energy maintenance through the activation and inhibition of master proteins of these pathways. The self-renewal of stem cells has been shown by an increase in the expression of stem cell markers like CD44 or CD133 and an increase in the ability to resist chemotherapeutic drugs, which maintains the survival of these cells within the tumor. In the blue circle, the main proteins that allow the interaction between these pathways are highlighted.

However, HBP is not the only nutrient-responsive pathway inside a cell: as shown in **Figure 5**, many complex interactions between the routes of HBP, AMPK, and mTOR combine nutritional signals to react to environmental changes. As a means of regulating cell function, the mammalian target of rapamycin (mTOR) and AMP-activated protein kinase (AMPK) pathways also engage in nutrient sensing and are significant factors in many pathologies. Crosstalk precisely adjusts the cellular response to nutrients between these pathways (15). Notably, the interaction between these nutrient-sensing pathways also significantly impacts stemness maintenance, as shown in **Figure 6**.

As mentioned before, AMPK functions as an energy sensor and is activated by a rise in the ratio of AMP-to-adenosine-5’-triphosphate (ATP). This molecular signal indicates that more energy is consumed in the cell than it is produced. Strong experimental evidence shows that AMPK negatively regulates the mTOR pathway, but O-GlcNAcylation of AMPK lowers its enzymatic activity resulting in growth promotion (95). Similar to how the AMP-to-ATP ratio represents cellular energy load, the UDP-GlcNAc abundance indicates the cell’s nutritional status because its synthesis involves glucose and metabolites derived from several major metabolic pathways. Zibrova et al. (96) provided a point of connection between AMPK and O-GlcNAc signaling; they demonstrated that the rate-limiting enzyme of the HBP signaling, glutamine/fructose-6-phosphate amidotransferase-1 (GFAT1), is a physiological substrate of AMPK. These authors also showed that the AMPK-mediated phosphorylation of GFAT1 at its residue Ser243 is inhibitory and occurs in response to physiological or small-molecule activators, thus leading to a reduction in the levels of cellular protein O-GlcNAcylation. Further work showed that AMPK-dependent phosphorylation of GFAT1 induces angiogenesis in endothelial cells (96).

The HBP pathway significantly participates in promoting stem cell marker expression. AMPK, on the other hand, can phosphorylate OGT, resulting in changes in the role of OGT. It has been shown that AMPK and OGT are substrates for each other and control each other’s activity in both non-malignant and malignant cells (**Figure 5**). For example, in HEK293T kidney cells, total cellular levels of O-GlcNAc regulate the activity of AMPK, with high levels of O-GlcNAc decreasing activation while increasing activation at low levels. These results are consistent with previous data indicating that the kinase mechanism is inhibited by AMPK O-GlcNAcylation (15, 97). In general, sustained O-GlcNAcylation is related to AMPK activation suppression, which could increase the activity of mTORC1 and increase the proliferation rate of cells. In colon cancer cells, Ishimura et al. (98) demonstrated that AMPK is OGlcNAcylated and that the increase in this protein modification diminishes AMPK phosphorylation resulting in mTOR pathway activation.

Concerning mTOR-HBP pathway interaction, Very et al. (99) showed that O-GlcNAcylation levels are controlled by mTOR in both normal and colon cancer cells. These authors also reported that down-regulation of O-GlcNAcylation by GFAT inhibition, or its up-regulation by OGA knockdown, decreases and increases, respectively, mTOR signaling activation. Moreover, Moloughney et al. (100) demonstrated that mTORC2 modulates the hexosamine biosynthetic pathway by promoting the expression of GFAT1, the key gate-keeper enzyme of the HBP. Therefore, HBP is likely to be closely regulated by nutrient levels and signaling molecules that regulate metabolism through GFAT1. These authors also identified Ser-243 in GFAT1as the site controlled by mTORC2. They found that in response to intracellular nutrient levels, mTORC2 regulates both the amplitude and duration of phosphorylation of GFAT1 at Ser-243 (100).

**Figures 5** and **6** show that the nutritional sensing pathways, such as AMPK, mTORC1, and O-GlcNAc, are full of feedback loops (15, 79, 83, 97, 99, 100) that enable a fine degree of control to be self-regulated and achieved. Several inputs interact in dynamic and complex ways. While decreased energy levels cause mTORC1 inhibition and AMPK activation, these conditions can also generate energy conservation through increased global O-GlcNAcylation.

## Concluding Remarks

Using nutrient-sensing pathways such as those regulated by HBP, mTOR, and AMPK, stem cells respond to nutritional cues, and the crosstalk between them is key to maintaining stemness (**Figure 6**).

There is still no agreement on cancer stem cells’ metabolic properties. Some studies show that they are predominantly glycolytic, and others indicate mitochondrial metabolism instead as their key energy source. But it is clear that CSCs are distinguished by a high plastic metabolism that enables them to withstand stressful conditions in the field.

Maintaining a continuous balance between nutrient availability and energy demand is necessary for all normal or malignant cells. Accumulating experimental evidence has shown that cancer-related glucose metabolism dysregulations interact with increased glucose flux *via* HBP, leading to high OGT expression and global levels of OGlcNAcylation. A key signaling molecule that regulates the metabolism of cells is mTOR. It operates through two distinct protein complexes, mTORC1 and mTORC2. Numerous studies have shown how, in response to nutrients supply, mTORC1promotes anabolic metabolism. By modulating the expression of GFAT1, the rate-limiting step in the HBP signaling route, mTORC2 controls the HBP. On the other hand, the mTOR pathway is negatively regulated by AMPK, an essential regulator of cellular and whole-body energy metabolism. This enzyme synchronizes metabolic processes to sense and balance nutrient availability with energy consumption. But cellular levels of O-GlcNAcylation control the activity of AMPK, with high levels of O-GlcNAc decreasing its activation, while low levels increase activation. Therefore, the interplay between HBP, mTOR, and AMPK pathways is involved in nutrient sensing to regulate cell activity and growth and, more importantly, in reacting to changes in the microenvironment of the tumor.

## Author Contributions

All authors listed have made a substantial, direct, and intellectual contribution to the work and approved it for publication. Specifically, their contributions were: Conceptualization, MR-F; investigation, AM-L, MCC-P and MR-F; figure design, AM-L and MCC-P; writing and editing, MR-F.

## Funding

This research was supported by grants from Universidad Nacional Autónoma de México (DGAPA-UNAM IV200220 and IN229420) and from CONACYT (FOSSIS 2017-289600).

## Conflict of Interest

The authors declare that the research was conducted in the absence of any commercial or financial relationships that could be construed as a potential conflict of interest.
